# Relationship of the Pro12Ala Polymorphism on the PPARy2 Gene With the Body Composition of Practitioners of Cyclic Exercises

**DOI:** 10.3389/fphys.2020.633721

**Published:** 2021-01-12

**Authors:** Raquel Suelen Brito da Silva, Darlene Camati Persuhn, Francisca Karinny Lemos Barbosa, Marizângela Ferreira de Souza, Klécia de Farias Sena, Matheus da Silveira Costa, Gisele Augusta Maciel Franca, Caroline Severo de Assis, Glêbia Alexa Cardoso, Alexandre Sérgio Silva

**Affiliations:** ^1^Laboratory of Applied Studies in Physical Training to Performance and Health-LETFADS, Department of Physical Education, Federal University of Paraíba, João Pessoa, Brazil; ^2^Associate Postgraduate Program in Physical Education – UPE/UFPB, Department of Physical Education, Federal University of Paraíba, João Pessoa, Brazil; ^3^Post Graduate Program in Nutrition Sciences, Federal University of Paraíba (PPGCN/UFPB), João Pessoa, Brazil; ^4^De Leve Clinic, João Pessoa, Brazil

**Keywords:** body composition, genetic polymorphism, PPARy2, energy expenditure, exercise

## Abstract

This study aimed to verify the association between the genotypic of the receptor gene activated by peroxisome proliferators gamma 2 (PPARy2) and the body composition and the specific indicators of adiposity in practitioners physical exercises, considering nutritional intake, age, and training load as influencing factors. It was conducted a cross-sectional study with 335 adults (47.9 ± 12.7 years, 138 men, body mass index/BMI = 27.0 ± 4.9 kg/m^2^), practitioners of aerobic exercises in cyclical modalities (running, walking and/or cycling, who spent 328.3 ± 193.6 kcal/day on physical training). The genotyping of the Pro12Ala polymorphism was performed using the PCR-RFLP technique and the body composition measured by bioimpedance (InBody 720). Energy expenditure was based on the compendium of physical activities and caloric intake was measured by 24 h recall questionnaire. The higher prevalence was for the Pro/Pro genotype (76.1% vs. 23.9% of Pro/Ala). Pro/Pro genotypic group showed significant higher mean values for body mass (BM) (*p* < 0.03 for men and *p* < 0.02 for women) and BMI (*p* < 0.00 for men and *p* < 0.02 for women) and %FAT (*p* < 0.00), waist-hip ratio (WHR) (*p* < 0.04), and visceral fat (VF) (*p* < 0.00) only in men compared to Pro/Ala. Higher frequency of Pro/Pro was observed in the category indicating BMI (*p* < 0.00 for men and *p* < 0.03 for women), WRH (*p* < 0.03 for men and *p* < 0.00 for women), and %FAT (*p* < 0.03) (in the latter case, only among men. It was also observed that the frequency of distribution of Pro/Ala in the eutrophic category of the BMI remained independent of all influencers, while WHR and %FAT were independent of the training load, but influenced by nutritional intake and age. In women, the frequency of Pro/Ala distribution at the lowest BMI and WHR values remained independent of all confounding variables. It is concluded that the Pro12Ala polymorphism in the PPARy2 gene consistently influences indicators of body composition and adiposity, regardless of the practitioners of physical training, but the relationship needs to be considered according to age and nutritional intake.

## Introduction

Obesity is considered a multifactorial phenomenon that involves environmental factors with genetic variants to result in this condition. On average, 40–70% of the inter-individual variability in body mass index and adiposity has been associated with these variants (Moustafa and Froguel, 2013; Zaitlen et al., 2013). Such variability is also observed in exercise practitioners (Sawyer et al., 2015), with some individuals losing body fat above average (responders), while others even gain body fat with a physical training program (non-responders), even considering protocols in which confounding factors, such as homogeneity between groups, nutrition and training load were well-controlled (Donnelly et al., 2013). This seems to indicate that differences related to biological individuality may be involved in this variation, with genetic aspects contributing to variations in body composition.

Regarding genetic influence, a gene which is associated with body composition is the peroxisome proliferator activated receptor gamma 2 (PPARy2) (Corrales et al., 2018; Cardoso et al., 2020). This nuclear receptor is strongly expressed in adipose tissue (Yao et al., 2015) and has a fundamental role in adipocyte differentiation, fatty acid metabolism and insulin sensitization. Although several polymorphisms have been reported in the PPARy2 gene, a very common polymorphism in this gene is Pro12Ala, which replaces a proline by alanine at position 12 (Becer and Çirakoglu, 2017). Studies dealing with the association of Pro12Ala with body mass have always shown conflicting results, with some showing that the presence of polymorphism is associated with an increased risk of obesity (Lapice and Vaccaro, 2014; Mansoori et al., 2015; Yao et al., 2015), while others show that it is associated with lower BMI and body mass (Deeb et al., 1998; Verhoef et al., 2014) or with no association (Ereqat et al., 2009; Mato et al., 2016; Wang et al., 2016).

One of the possible causes of that conflict is that previous studies do not consider nutritional intake (Ebbeling et al., 2018), age (Palmer and Kirkland, 2016), level of physical activity (Campbell et al., 2018) or systematic physical training (Westerterp, 2018), factors known to influence body composition. However, the relationship of the Pro12Ala polymorphism already reported indicates the influence of this gene on body composition. In addition, in these previous studies, the variables considered were only body weight and BMI, while modern devices available incorporate markers more directly associated with body fat deposits (percentage of fat, absolute weight of body fat and visceral fat), and others that in addition to BMI have been considered relevant in populational studies (waist-to-hip ratio).

Thus, we tested the hypothesis that genotypic groups of the Pro12Ala polymorphism of the PPARy2 gene from practitioners of systematic physical training show differences in body composition variables (body mass, BMI) and more specific variables for body adiposity (fat percentage, waist hip ratio, visceral fat), and that this possible genotypic influence may be modulated by nutritional intake, age and training load.

## Materials and Methods

### Participants

It was conducted a cross-sectional study involving 335 practitioners of aerobic exercises in cyclical modalities (walking/running and/or cycling). This sample was representative of the population of a city in Brazil (South America), being made of a conglomerate by adopting a map of the city health department which divides the city geographically into five districts. From each district, two public environments that used to practice physical exercises were randomly selected.

The inclusion criteria adopted were: both sexes, being between 18 and 59 years of age, practicing cyclic exercise (walking/running and/or cycling) for at least 1 year and not having interrupted training for more than 2 weeks in last 3 months; when practicing a sport other than the exercises in question, have not exceeded 20% of this weekly load; not having followed any protocol (nutritional or pharmacological) for loss of body mass in the last 12 months; reporting that they are maintaining the same dietary pattern in the last 12 months. Volunteers who gave up any of the steps provided for in the protocol were excluded.

### Recruitment of Participants and Evaluations

The visits were carried out during the hours of greatest flow of practitioners (from 5:00 to 7:00 am and from 4:00 to 7:00 pm). The individuals were approached at the time they were practicing the physical exercise to guarantee the randomness of the research. The questions asked at the training site were intended to make a general assessment of these inclusion criteria. Those ones who met the sample inclusion criteria were scheduled for the interview and data collection.

### Assessment of Food and Dietary Consumption

Assessment of food intake was performed using the 24 h recall. The instrument was applied by two trained nutritionists, taking into account 3 days referring to the food consumption of 2 days a week and 1 weekend (triplicate). After that, the mean values for the analysis of the macronutrients of the food (carbohydrate, protein, and fat) were taken in absolute and relative terms, and also the values of ingestion of each of them relative to the participant’s body mass (Gibson, 1990), using the Avanutri software version 4.0 (AVANUTRI-RJ, Brazil).

### Body Composition Assessment

Body composition was assessed using Inbody octapolar bioimpedance (model 720, Biospace, Korea). This device was previously validated against dual-energy X-ray absorpometry (DEXA) as gold standard for analyzing body composition (McLester et al., 2020). Body mass (BM), body fat percentage (%FAT), fat mass (FM), visceral fat (VF), waist-hip ratio (WHR), and skeletal muscle mass (SMM) were measured. For this assessment, the volunteers followed some pre-test procedures: being fasted, not having done moderate to high intensity exercise in the 12 h before the assessment, not having consumed alcohol 48 h before the test, not having coffee, not wearing metal jewelry or dental implants with metal during the evaluation and wearing light clothing during the procedure. The height measurement (m) was verified in a portable analog stadiometer of the Sanny Medical^®^ brand with precision of 0.1 cm, being evaluated following the recommendation of Tritschler (2003). For the BMI, a cutoff point of < 25 − ≥ 25 kg/m^2^ was used (World Health Organization (WHO), 2020), WHR was used a cutoff point of < 0.90 − ≥ 0.90 cm for men and < 0.85 − ≥ 0.85 cm for women (World Health Organization (WHO), 2011) and %FAT according to the age group (American College of Sports Medicine (ACSM), 2019).

### Training Load Profile

The training load was based on the frequency, duration and speed adopted in cyclic exercises (walking/running and/or cycling). When the volunteer had accurate information about the distance traveled daily and the time spent on that distance, the speed was calculated based on this information. In opposite cases, speed and distance covered were assessed by GPS devices (Nike Run). The Physical Activities Compendium proposed by Ainsworth et al. (2000) to estimate energy expenditure. Based on these data, the calculation of caloric expenditure (McArdle et al., 1998) was performed using the following formula: kcal = (metabolic equivalent (MET) of activity × body mass (kg)/60) × activity time (min). The sum of the caloric expenditure of all training sessions was divided over 7 days a week.

The volunteers carried out the training in a city where the temperature varies between 24 and 33°C throughout the year, without periods of intense rain, so the climatic factor was not a barrier to this practice in most part of the year.

### Genotyping

Genomic DNA was extracted using oral cells (mouthwash) and made according to the method of Aidar and Line (2007). Genotyping of the Pro12Ala polymorphism (PPAry2) was performed by the PCR-RFLP technique and the polymorphism was amplified using the primers: (5′-GCCAATTCAAGCCCAGTC-3′-sense and 5′-GATATGTT GGAGAGAGGGTATCAGTGAAGGAATCGCTTTCCG-3′-antisense).

**FIGURE 1:**
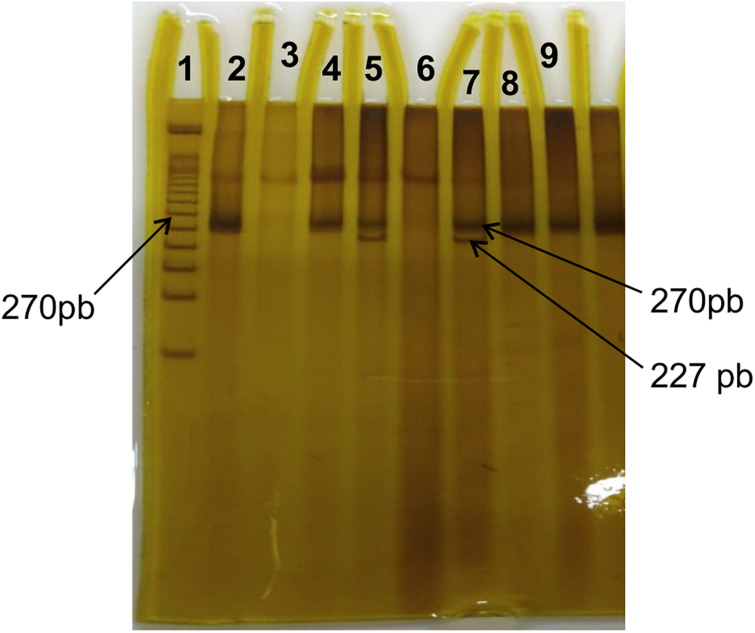
Polyacrylamide gel stained with silver nitrate from the genotyping sample for Pro12Ala. Line 1 corresponds to the 50 bp ladder. Lines 2, 3, 4, 6, 8, and 9 show the non-fragmented Pro/Pro genotype (size 270 bp); lines 5 and 7 show fragments with sizes of 270 and 227 bp resulting from the restriction site, with the replacement of Proline-Alanine.

The PCR products were digested at 37°C, for 3 h, using the restriction enzyme BstU-I (Biolabs, New England/United States). After that, they were submitted to an electrophoretic run in polyacrylamide gel at a concentration of 15%, so that the bands were developed with silver nitrate. The products expected after digestion were 270 bp for homozygous (Pro/Pro), 227 and 43 bp for homozygous (Ala/Ala) and 270, 227, and 43 bp for heterozygous (Pro/Ala) (Figure 1).

### Data Analysis

Normality and homogeneity were assessed using the Kolomogorov—Smirnov test and the Levene test, respectively. Descriptive analyzes were frequency distribution, mean and standard deviation or median, and confidence interval. An independent *T*-test was performed (or non-parametric equivalent) to assess differences between the characteristics of the two groups (Pro/Pro and Pro/Ala) and a Pearson chi-square test was conducted to verify differences of the genotypic groups for variables for which there are recognized cutoff points BMI, adopting the cutoff point of < 25 − ≥ 25 kg/m^2^ (World Health Organization (WHO), 2020); WHR according to the cutoff point of < 0.90 − ≥ 0.90 cm for men and < 0.85 − ≥ 0.85 cm for women (World Health Organization (WHO), 2011) and %FAT according to the age group (American College of Sports Medicine (ACSM), 2019). After that, a binary logistic regression test was performed considering nutritional intake, age and load training as possible confoundings factors in the associations between genotype and body composition. The significance level adopted was *p* < 0.05, and the data were tabulated in SPSS Statistics software (v. 25, IBM SPSS, Chicago, IL).

### Ethical Aspects

The study was approved by the Research Ethics Committee of the Health Sciences Center of the Federal University of Paraíba (CEP/CCS/UFPB), under protocol no. 0320/15 and CAAE: 45903215.4.0000.5188. And the participants, after being informed about the risks and benefits, signed the free and informed consent term, according to Resolution 466/12 of the National Health Council (CNS- Brasil).

## Results

In 335 collected participants, a prevalence of 23.9% (*n* = 80) of the Pro12Ala genotype was observed, with 76.1% (*n* = 255) of Pro/Pro and no occurrence of Ala/Ala. Among men (*n* = 138), the frequency of Pro12Ala was 27.5%, and in women (*n* = 197), 21.3% (Table 1). Pro/Pro and Pro/Ala groups showed similar energy expenditure in physical training, as well nutritional behavior for total caloric and macronutrient intake, both in men and women, with normocaloric, normoglycide, normoprotein, and normolipidic food patterns (Table 1).

**TABLE 1:** Body composition profile according to the allele frequency distribution in men and women.

	Men (*n* = 138)			Women (*n* = 197)		
Variables	Pro/Pro *n* = 100	Pro/Ala *n* = 38	*p*	Pro/Pro *n* = 155	Pro/Ala *n* = 42	*p*
	**(72.5%)**	**(27.5%)**		**(78.7%)**	**(21.3%)**	
Age (years)	44.9 ± 12.3	41.5 ± 10.9	0.13	51.0 (48.6–52.2)	53.0 (45.2–53.6)	0.54
BM (kg)	77.8 ± 14.2	72.8 ± 10.9	0.03^∗^	68.4 ± 12.7	63.8 ± 9.9	0.02^∗^
BMI (kg/m^2^)	26.7 ± 4.6	24.5 ± 3.9	0.00^∗^	28.1 ± 5.2	26.2 ± 3.9	0.02^∗^
%FAT	23.7 (22.2–26.0)	18.2 (16.7–22.7)	0.00^∗^	38.4 (37.2–39.7)	37.1 (34.8–39.5)	0.25
FM (kg)	19.5 ± 10.0	15.0 ± 8.7	0.00^∗^	26.8 ± 9.7	23.9 ± 7.7	0.06
WHR (cm)	0.89 ± 0.08	0.86 ± 0.07	0.04^∗^	0.91 ± 0.05	0.89 ± 0.07	0.11
VF (cm^2^)	80.2 (71.7–88.5)	52.6 (49.0–73.0)	0.00^∗^	101.4 ± 28.2	93.4 ± 28.5	0.10
SMM (kg)	32.9 ± 5.4	32.5 ± 4.2	0.70	22.6 ± 3.0	21.9 ± 3.3	0.20
Exercise charge (kcal/day)	394.2 ± 146.2	419.1 ± 136.8	0.36	265.2 ± 118.6	260.2 ± 126.1	0.81
Total kcal	2151.8 ± 729.6	2254.7 ± 629.9	0.31	1466.7 (1452.2–1602.9)	1461.0 (1325.2–1619.6)	0.76
Kcal/kg	28.9 ± 12.2	31.7 ± 10.0	0.11	22.9 ± 8.0	23.3 ± 8.1	0.79
CBO (g/kg/day)	3.8 ± 1.8	4.1 ± 1.6	0.17	3.0 ± 1.2	3.0 ± 1.3	0.59
FAT (g/kg/day)	0.8 (0.7–0.9)	0.9 (0.8–1.1)	0.08	0.6 (0.6–0.7)	0.6 (0.6–0.8)	0.83
PRO (g/kg/day)	1.5 ± 0.7	1.8 ± 0.9	0.06	1.1 (1.0–1.2)	1.1 (1.0–1.4)	0.39

Data are presented as mean ± SD or median with confidence interval. Legend: BM, body mass; BMI, body mass index; %FAT, body fat percentage; FM, fat mass; WHR, waist-to-hip ratio; VF, visceral FAT; SMM, skeletal muscle mass; CBO, carbohydrate; FAT, fat; PRO, protein. (Student’s t-test for independent samples or Mann-Whitney test). *p < 0.05.

It was found that Pro/Pro homozygous men had a mean BMI compatible with overweight and significantly higher than Pro/Ala, who in turn had a mean compatible with eutrophy. Significant differences were also seen for BM, %FAT, FM, WHR and VF, in which subjects Pro/Pro had always higher values compared to genotyped Pro/Ala. The Pro/Pro women also showed a higher level of obesity, but statistical differences in relation to Pro/Ala were noted only for BMI and BM, so that measures associated with body fat (%FAT, FM, WHR, and VF) were similar between the two genotypic groups of women. Details of this data can be seen in Table 1.

For variables that allow categorization according to established cutoff points (BMI, WHR, and %FAT), differences in the frequency of distribution of people with ideal values and indicators of obesity between the Pro/Pro vs. Pro/Ala genotypes were tested. Statistical differences were found with a higher frequency of Pro/Pro subjects in the category above the normal to obesity indicators and higher frequency of Pro/Ala in the categories they classify as normal (Table 2). Among men, there was a significantly higher frequency in the distribution of Pro/Pro participants in the category of higher BMI (odds ratio for eutrophy in the Pro/Ala of 25% compared to the Pro/Pro), WHR (odds ratio for eutrophy in the Pro/Ala of 44% compared to the Pro/Pro) and %FAT (odds ratio for eutrophy in the Pro/Ala of 25% compared to the Pro/Pro). For women, there was a significant difference with a greater distribution of Pro/Pro in the more obese profile for BMI (odds ratio for eutrophy in the Pro/Ala of 46% compared to the Pro/Pro) and WHR (odds ratio for eutrophy in the Pro/Ala of 30% compared to the Pro/Pro), and no significant difference for %FAT.

**TABLE 2:** Frequency distribution of genotypes in the subgroups according to BMI, WHR, and %FAT.

Genotype	Men (*n* = 138)			Women (*n* = 197)		
	BMI < 25	BMI ≥ 25	*p*	BMI < 25	BMI ≥ 25	*p*
Pro/Pro	38 (38.0%)	62 (62.0%)	0.00^∗^	40 (25.8%)	115 (74.2%)	0.03^∗^
Pro/Ala	27 (71.1%)	11 (28.9%)		18 (42.9%)	24 (57.1%)	
Odds ratio	**0.25 (0.11–0.56)**			**0.46 (0.22–0.94)**		

	**WHR < 0.90**	**WHR ≥ 0.90**		**WHR < 0.85**	**WHR ≥ 0.85**	

Pro/Pro	52 (52.0%)	48 (48.0%)	0.03^∗^	17 (11.0%)	138 (89.0%)	0.00^∗^
Pro/Ala	27 (71.1%)	11 (28.9%)		12 (28.6%)	30 (71.4%)	
Odds ratio	**0.44 (0.19–0.98)**			**0.30 (0.13–0.71)**		

	**%FAT ideal**	**%FAT above ideal value**		**%FAT ideal**	**%FAT above ideal value**	

Pro/Pro	46 (46.0%)	54 (54.0%)	0.03^∗^	17 (11.0%)	138 (89.0%)	0.57
Pro/Ala	25 (65.8%)	13 (34.2%)		4 (9.5%)	38 (90.5%)	
Odds ratio	**0.46 (0.21–1.00)**			**1.09 (0.34–3.46)**		

Data are absolute and relative frequency. BMI, body mass index (cutoff point of < 25 − ≥ 25 kg/m^2^, according to World Health Organization (WHO), 2020); WHR, waist-to-hip ratio (cutoff point of < 0.90 − ≥ 0.90 cm for men and < 0.85 − ≥ 0.85 cm for women according to, World Health Organization (WHO), 2011); %FAT, body fat percentage (cutoff depends on age, according to the American College of Sports Medicine (ACSM) (2019) protocol). Pearson Chi-square test. *p < 0.05.

A regression model was performed to verify whether age, energy expenditure of physical training (average daily caloric expenditure resulting from the volume × intensity adopted by individuals in the training sessions) and nutritional intake (carbohydrates, fats and proteins normalized by body mass) would influence the more eutrophic profile of the Pro/Ala group compared to the Pro/Pro group.

It was observed that the higher frequency of Pro/Ala men in the eutrophic classification in relation to BMI remained regardless of age, energy expenditure in training sessions, and nutritional intake (beta data and odds ratio are shown in Table 3). Meanwhile, the WHR association remained independent of the energy expenditure in physical training (B = −0.886 ± 0.42; *p* < 0.03; odds ratio = 0.41: 0.18–0.44 95%, confidence interval), but lost statistical significance toward caloric intake and age (data in Table 3). Something similar occurred for %FAT, in which the association remained independent of the energy expenditure in the training sessions (B = 0.819 ± 0.40; *p* < 0.04; odds ratio = 0.44: 0.19–0.98, 95% confidence interval), but lost significance in the face of nutritional intake and age (data in Table 3).

**TABLE 3:** Logistic regression model verifying the influence of energetic demand on physical training, intake of macronutrients (carbohydrates, proteins, and fats) and age on the association between Pro/Ala polymorphism in PPARy2 and body composition of men and women.

	Men (*n* = 138)			Women (*n* = 197)		
	B	*p*	OR	B	*p*	OR
**BMI**						
BMI	−1.384 ± 0.48	0.00^∗^	0.25 (0.09–0.64)	−0.870 ± 0.42	0.04^∗^	0.41 (0.18–0.96)
EXERCISE	0.00 ± 0.00	0.80	1.00 (0.99–1.00)	0.000 ± 0.00	0.76	1.00 (0.99–1.00)
CBO (g/kg)	−0.134 ± 0.21	0.54	0.87 (0.57–1.34)	−0.094 ± 0.18	0.61	0.91 (0.63–1.31)
FAT (g/kg)	0.903 ± 0.79	0.25	2.46 (0.52–11.69)	−0.399 ± 0.69	0.56	0.67 (0.17–2.64)
PRO (g/kg)	−0.031 ± 0.45	0.94	0.97 (0.40–2.34)	0.376 ± 0.43	0.38	1.45 (0.62–3.41)
Age	−0.005 ± 0.01	0.77	0.99 (0.95–1.03)	0.003 ± 0.01	0.86	1.00 (0.97–1.03)
**WHR**						
WHR	−0.707 ± 0.46	0.12	0.49 (0.20–1.21)	−1.239 ± 0.46	0.00^∗^	0.29 (0.11–0.72)
EXERCISE	0.001 ± 0.00	0.67	1.00 (0.99–1.00)	−0.001 ± 0.00	0.73	0.99 (0.99–1.00)
CBO (g/kg)	0.000 ± 0.20	0.99	1.00 (0.66–1.49)	−0.123 ± 0.19	0.51	0.88 (0.60–1.28)
FAT (g/kg)	1.056 ± 0.76	0.16	2.87 (0.64–12.89)	−0.137 ± 0.68	0.84	0.87 (0.22–3.34)
PRO (g/kg)	−0.097 ± 0.44	0.82	0.90 (0.38–2.16)	0.357 ± 0.44	0.41	1.42 (0.60–3.39)
Age	−0.010 ± 0.01	0.57	0.99 (0.95–1.02)	0.002 ± 0.01	0.91	1.00 (0.97–1.03)
**%FAT**						
%FAT	−0.613 ± 0.43	0.16	0.54 (0.22–1.28)	0.259 ± 0.62	0.67	1.29 (0.38–4.40)
EXERCISE	0.001 ± 0.00	0.72	1.00 (0.99–1.00)	−0.001 ± 0.00	0.73	0.99 (0.99–1.00)
CBO (g/kg)	−0.017 ± 0.20	0.93	0.98 (0.65–1.47)	−0.012 ± 0.18	0.94	0.98 (0.69–1.41)
FAT (g/kg)	1.054 ± 0.75	0.16	2.86 (0.65–12.65)	−0.059 ± 0.67	0.93	0.94 (0.25–3.55)
PRO (g/kg)	0.020 ± 0.43	0.96	1.02 (0.43–2.37)	0.410 ± 0.43	0.34	1.50 (0.64–3.52)
Age	−0.009 ± 0.01	0.62	0.99 (0.95–1.02)	−0.008 ± 0.01	0.60	0.99 (0.96–1.02)

Regression model: Genotype (Pro/Pro and Pro/Ala), as a dependent variable. Energetic demand in physical training, Macronutrients intake (carbohydrate, protein, and fat) and age as independent variables. A model with total caloric intake was also run separately, to avoid collinearity between the variables, but the results were similar. BMI, body mass index (< 25 − ≥ 25 kg/m^2^, World Health Organization (WHO), 2020); WHR, waist-to-hip ratio (men < 0.90 − ≥ 0.90 cm; women < 0.85 − ≥ 0.85 cm, World Health Organization (WHO), 2011); %FAT, body fat percentage [cut-off point depends on age according to American College of Sports Medicine (ACSM) (2019)]; CBO, carbohydrate; FAT, fat; PRO, protein. *p < 0.05.

Among women, the significant higher frequency of BMI compatible with eutrophy among Pro/Ala remained regardless of energy expenditure in training, nutritional intake and age (beta data and odds ratio shown in Table 3). The same occurred for the higher frequency of Pro/Ala women with WHR classified as normal compared to women with values considered above normal limits. The absence of association between the genotype and the frequency of distribution in relation to the %FAT that had already been demonstrated in the chi square test (Table 2), remained when considering the confusing energy expenditure in training sessions, nutritional intake, and age (Table 3).

## Discussion

This study showed that people genotyped with Pro/Pro and Pro/Ala in the PPARy2 gene show differences in body composition both in the mean values of body weight, BMI, %FAT, fat mass, WHR, and in the frequency of distribution according to the points of normality cut for BMI, WHR, and %FAT, with indication of normal weight always present in the Pro/Ala group. These differences are somewhat different according to sex and are independent of sex, age and energy expenditure in physical training for BMI between men and women and WHR in women.

Previous studies on the relationship between Pro12Ala and body composition have conflicting results. About this, we had assumed that one of the causes of this inconsistency could be the fact that such data take into account only body mass and/or BMI, which was even confirmed through our findings that BMI is associated with investigated genotypes, corroborating with the studies of Deeb et al. (1998) and Verhoef et al. (2014). However, in addition to this variable, we demonstrated that those more directly related to body fat, such as %FAT and visceral fat, made the statistical demonstration of association between the genotype and the body composition of the population studied more consistent.

Another possibility to the controversy is that most authors do not consider the differences between the sexes. This is an important issue considering that body composition is different between men and women. Bredella (2017) explains that women have more fat mass than men, while men have more lean mass. In fact, our data showed that, for variables in which differences in body composition were seen between the genotypic groups in men, the same did not occur in women, both when comparing the mean and frequency of distribution.

The adjustment for nutritional intake and age seems to be the factor that most clarifies the conflicts noted in previous studies. Considering only the variables adopted in these previous publications (BMI and WHR), our data showed that the associations found between the frequency of distribution of genotypes for %FAT did not persist in women and WHR did not persist among men when adjustment was made by nutritional intake. Meanwhile, %FAT has dissipated among men in adjusting for age, as has BMI among women. So, our data clarify that conflicts of results may be and are better explained by considering potential confounding factors for the relationship of the genotype with body composition.

Unlike previous studies, the present investigation was done with a population that is adept at systematic physical training, while in the others as people were sedentary or did not adhere to a systematic physical training program. Although with several methodological similarities (genotypic groups, statistical test of distribution frequency), our data answer a question different from previous studies. While they verified whether the polymorphism in PPARy2 is a determinant of body composition in a population, our data reflect a possible influence of Pro12Ala in the relationship between physical training and body composition. Although indirectly, these findings may contribute to clarify one of the most pertinent current issues in the literature. The literature has shown that exercise has limitations in the weight loss process (Boutcher and Dunn, 2009; Johns et al., 2014; Washburn et al., 2014; Pontzer et al., 2016). Although some explanations have been made in an attempt to explain this limitation, such as adaptive thermogenesis and physiological factors (e.g., oxidative stress, hyperinsulinemia) (Boutcher and Dunn, 2009), the possibility that some genetic aspect may explain this resistance to weight loss cannot be ruled out. Therefore, our data cast a perspective that Pro/Pro homozygous people may be more resistant to exercise-induced weight loss.

This evidence needs to be investigated with a randomized controlled trial study to verify this effect directly on weight loss responses from a training program. In fact, our laboratory tested the relationship of the Pro12Ala and found no influence of this polymorphism on the magnitude of weight loss found in its genotypic groups (Cardoso et al., 2020). However, when we analyzed the polymorphism in PPARα intron 7G/C (rs4253778), we found a significant difference in the weight loss response with people genotyped with CA + AA losing 35.7% more than those genotyped with CC (still unpublished data).

The place where the research was carried out would be ideal for carrying out this intervention study, due to the high prevalence found of people with Ala (23.9%). This value was much higher when compared to the prevalence of this genetic variant in previous studies, such as 10% in Native Americans (Mori et al., 2001), 8.7% in Arabs (Al-Jarallah et al., 2011) and 9 and 12.4% in Brazilians (Caramori et al., 2003; Rodrigues et al., 2018). On the other hand, one limitation of the present study is the analysis of only one polymorphism. It is known that the coexistence of other genetic variants related to energy metabolism such as melanocortin receptor (MC4R), obesity protein and associated fat mass (FTO), pro-opiomelanocortin (POMC), interleukin 6 (IL6), uncoupling protein 1 (UCP1), perilipine 5 (PLIN 5) agouti-related peptide (AGRP), leptin (LEP) may increase the influence of Pro12Ala on body composition. In addition, our data showed that only the genetic profile alone is not able to explain body composition, since regression data showed that nutritional factors and age are potentially involved in body composition. Even if other polymorphisms are investigated in the future, these factors need to be considered together so that the size of the genetic influence on the body composition of a population can be more precisely determined.

Finally, we must point out that the analysis technique was bioimpedance, while DEXA is considered the gold standard for this measure (McLester et al., 2020). However, the device we use is the manufacturer’s first line and has been validated against DEXA with a correlation coefficient ≥0.98 for %FAT and FM and ≥0.99 for fat-free mass (McLester et al., 2020). In fact, even previous versions of this device have been accepted in scientific publications, including in intervention studies (Irandoust and Taheri, 2015).

This brings an advance in scientific research and clinical practice, because from these data, it is expected that in addition to the genetic profile, intervening variables such as nutritional intake and age will be considered in people who practice physical exercise, but do not have adequate body composition.

## Conclusion

Finally, this study sheds light on a conflict in the literature regarding the relationship between the Pro12Ala polymorphism in the PPARy2 gene and body composition indicators, since in our findings the Pro/Pro genotyped groups had higher mean values and frequency distribution in body composition, with normality indication always for the Pro/Ala group. In addition, the regression showed that Pro12Ala consistently influences these variables, regardless of the practice of physical training, age and nutritional intake must be taken into account.

## Data Availability Statement

The original contributions presented in the study are included in the article/supplementary material, further inquiries can be directed to the corresponding author/s.

## Ethics Statement

The studies involving human participants were reviewed and approved by the Research Ethics Committee of the Health Sciences Center of the Federal University of Paraíba (CEP/CCS/UFPB). The patients/participants provided their written informed consent to participate in this study.

## Author Contributions

RS and AS conceived the idea of the study, contributed to the writing, editing, and approval of the final version of the manuscript. DP, FB, MS, KS, MC, GF, CA, and GC conceived the editing the manuscript and approved the final draft of the manuscript. All authors contributed to the article and approved the submitted version.

## Conflict of Interest

The authors declare that the research was conducted in the absence of any commercial or financial relationships that could be construed as a potential conflict of interest.
